# Fascial Manual Medicine: The Concept of Fascial Continuum

**DOI:** 10.7759/cureus.82136

**Published:** 2025-04-12

**Authors:** Bruno Bordoni, Allan R Escher

**Affiliations:** 1 Physical Medicine and Rehabilitation, Foundation Don Carlo Gnocchi, Milan, ITA; 2 Oncologic Sciences, University of South Florida Morsani College of Medicine, Tampa, USA; 3 Anesthesiology/Pain Medicine, H. Lee Moffitt Cancer Center and Research Institute, Tampa, USA

**Keywords:** chiropractic, fascia, fasciae, fascintegrity, manual therapy, myofascial, osteopathic, osteopathy, physiotherapy, quantum biology

## Abstract

Fascial tissue ubiquitously pervades the body system, becoming the target of many disciplines that use manual techniques for patient treatment. It is a much-debated topic as there is currently no univocal definition among different authors. Due to the non-discontinuity of the fascia, we can speak of a fascial continuum; this principle is the basis of the osteopathic perspective. This vision, which seems banal, is not always applied in manual fascial medicine, where, often, it is conditioned by a reductionist (layers) and mechanistic (compartments) approach, forgetting that the body is not a machine but an organism. This continuity teaches that manual treatment does not only reverberate in the area where the operator’s hands rest but creates a series of local and systemic adaptations. This narrative review revises the concept of the fascial continuum by highlighting that fascia is a tissue system (different tissues working in harmony), multi-organ (capable of behaving like an organ), whose macroscopic functional expression (movement) and microscopic (with cellular adaptations) derives from a nanoscopic coherence (electromagnetic behaviors). This means that the body acts as a unit, and makes the manual approach never local but always systemic. The aim of the article is to highlight the fact that the fascial continuum is a single biological entity (solid and fluid), and that manual fascial medicine does not approach a single segment, but the entire person.

## Introduction and background

The human body acts as a unit, expressing in the macroscopic the functions that derive from the coherence of nanoscopic structures, such as phonons and photons [1,2]. Observing the human body only from a macroscopic view is a short-sighted act, as well as considering that the manual action of the clinician on the patient will have an impact only on the clinician's work area [3,4].

A simple caress will be registered by the nervous system, peripheral and central, activating all the body components, starting from the mechanosensory afferents (C-tactile afferents), traveling through the spinothalamic pathways, and reaching the limbic areas, the cognitive and behavioral cortical areas [5]. This information (from the caress) will stimulate hormonal and neuropeptide responses (oxytocin, dopamine, opioid system), will influence the general metabolism (reduction of heart rate and increase of vagal tone), the perception of pain, and the motor aspect [6].

When we stimulate a specific area of the musculature to stimulate hypertrophic processes, the macroscopic system is involved (arthro-muscular movement), and all internal systems are activated (neural, endocrine, immune, enteric, cardio-respiratory); the previous systems will be responsible for microscopic responses (hormones, neuropeptides, cytokines, contractile proteins, satellite cells, growth factors, different ions, macrophages, and more), and adaptations at the nanoscopic/quantum level (vibrations deriving from actomyosin or spontaneous oscillatory contraction of sarcomeres, emission of phonons and photons) [7-14]. The body is an absolute continuum.

We could also look at the body as a set of interconnected and interdependent networks. It is enough to think of the neural, lymphatic, and vascular networks, which are intertwined, interacting, and pervading the entire body. Furthermore, the interstitial space itself is distributed ubiquitously throughout the body system, whose interior is filled with fluids that transport numerous biochemical substances and interface with all tissues; these fluids generate pressure and mechanotransductive information regulate the function and morphology of body cells, protecting them from non-physiological mechanical stress [15-19]. There is no discontinuity from a functional point of view.

An osteopathic manual approach on the occipital area is able to mitigate blood pressure in patients with hypertension, as the manual technique acts on the autonomic system [20].

A pilot study reported that working manually with an osteopathic approach (rib heads at the costotransverse articulation) can modulate the systemic function of the autonomic system (decrease of the sympathetic system), measurable by the decrease of salivary α-amylase [21]. Gentle osteopathic treatments, regardless of the treated area, can modulate (decrease) the level of cytokines and leukocytes, with a change in the immunological profile [22]. Manual techniques used by clinicians, osteopaths, chiropractors, physiotherapists, and other health professionals do not act only on the specific body area of treatment, but always on the whole body as the human being functions as a continuum and not as a machine with compartments or layers. The body must be seen as a biological and not a mechanical structure. Let us recall the example of manual treatment on the occipital area which gives a cardiovascular (therefore, systemic) response [20].

Most tissues can be included in the definition of fascia, and fascia is the target of many disciplines that use manual techniques for patient treatment [23]. This narrative review revises the concept of the fascial continuum considering information from different disciplines, highlighting that from the functional point of view, the body cannot be traced back to a reductionist (layers) and mechanistic (compartments) vision. Science (a set of knowledge) should be looked at from a gnoseological perspective, that is, episteme (scientific knowledge) is not valid only thanks to acquired knowledge but is valid knowing that scientific information is not immutable and that it can depend on the ability to understand the same knowledge; this determines the continuous evolution of knowledge and science or epistemic evolution.

## Review

Staying in the past

In Western medicine, Helkiah Crooke, anatomist, (1615) was the first anatomist to use the term “fascia”; Dr. Astley Paston Cooper, surgeon and anatomist, was the anatomist who used the term “fasces” in his writings, identifying membranous structures that covered muscle [24]. The latter scholar laid the foundation for the classification of how fascia is understood in this century, writing that fascial tissue was found throughout the body [25]. In the eighteenth century, fascia was understood as a loose, membranous structure that covers and connects musculature, building a scaffolding to hold the skeletal muscle in place [24]. Samuel Foart Simmons, physician, and anatomist, in 1780 was the first in anatomy writings to try to observe fascia from a microscopic point of view, describing fascia as a network with water inside [24]. In the 19th century, the term fascia was associated with the anatomical area of origin, the depth, the shape, the thickness of the tissue, the possible functions, macroscopic characteristics, and with respect to the different connections with which the fascia interfaced [24]. In the eighteenth and nineteenth centuries, the fascia was described as an organ [24,25].

In the twentieth century, the classification of fascia took on a purely topographical connotation from anatomists (Federative Committee on Anatomical Terminology, International Federation of Associations of Anatomists), and classification with respect to the depth of detection as surgeons needed to know the presence of one tissue compared to another [24]. In the twenty-first century, fascia also became of interest to clinicians who employ manual treatments, such as osteopaths, chiropractors, physiotherapists, massage therapists, and other health professionals. The Fascia Research Congress (2007) began a journey to define and classify fascial tissue, under the impetus of scholars and researchers who were orienting interest toward manual medicine [24]. The organizers of this congress proposed a nomenclature of what the term fascia means, the most recent of which is from 2019:

“The fascial system consists of the three-dimensional continuum of soft, collagen-containing, loose and dense fibrous connective tissues that permeate the body. It incorporates elements such as adipose tissue, adventitia, and neurovascular sheaths, aponeuroses, deep and superficial fasciae, epineurium, joint capsules, ligaments, membranes, meninges, myofascial expansions, periosteum, retinacula, septa, tendons, visceral fasciae, and all the intramuscular and intermuscular connective tissues including endo-/peri-/epimysium. The fascial system interpenetrates and surrounds all organs, muscles, bones and nerve fibers, endowing the body with a functional structure, and providing an environment that enables all body systems to operate in an integrated manner” [26].

The fascial system is seen from a mechanical point of view, where the fascial layers represent the compass to orient the manual treatment [27,28]. But, “in truth, there are no separate sheets” [29].

Fascial manual medicine does not need to know the layers, as the whole body encounters manual treatment; the whole fascial continuum is in contact [26]. The body system, being an absolute and functional continuity, is always aware of what is happening inside the body; this awareness allows for local and systemic adaptations to be created, independently of the tissue layers.

The fascia is considered a sensory organ, as the connective tissue that covers and interpenetrates the musculature is rich in mechanoreceptors [30,31]. But do all the structures that fall under fascia have mechanoreceptive properties and structures (for example fat)? If for the surgeon or anatomist’s vision, the body is organized in layers, what is the fat layer, or the periosteum layer, and others?

There are some inconsistencies regarding the consideration of fascia as layers or thinking of fascia only as connective tissue involving muscles and bones, without including other tissues. Also, to give another example highlighting the confusion on this topic, fat is found in different areas of depth in the human body (cardiac fat, subcutaneous fat); therefore, is this tissue part of the superficial and/or deep layer? There is a difference in depth between the periosteum of the femur and the radial bone.

Is it so easy to find the layer to apply a manual technique? Is it possible to palpate the deep layer rather than the endomysium? Is it so easy to discern the superficial layer from the perimysium? Without an ultrasound scanner and the clinical habit of using this instrument correctly, no one can say that the operator who is performing palpation or a manual approach is exactly where the operator would like to be with his hand [32-37]. Furthermore, there are many anatomical variables, and being sure that the manual treatment is always on the body structure that one intends to work on, without instrumental help, is a gamble.

**Figure 1:**
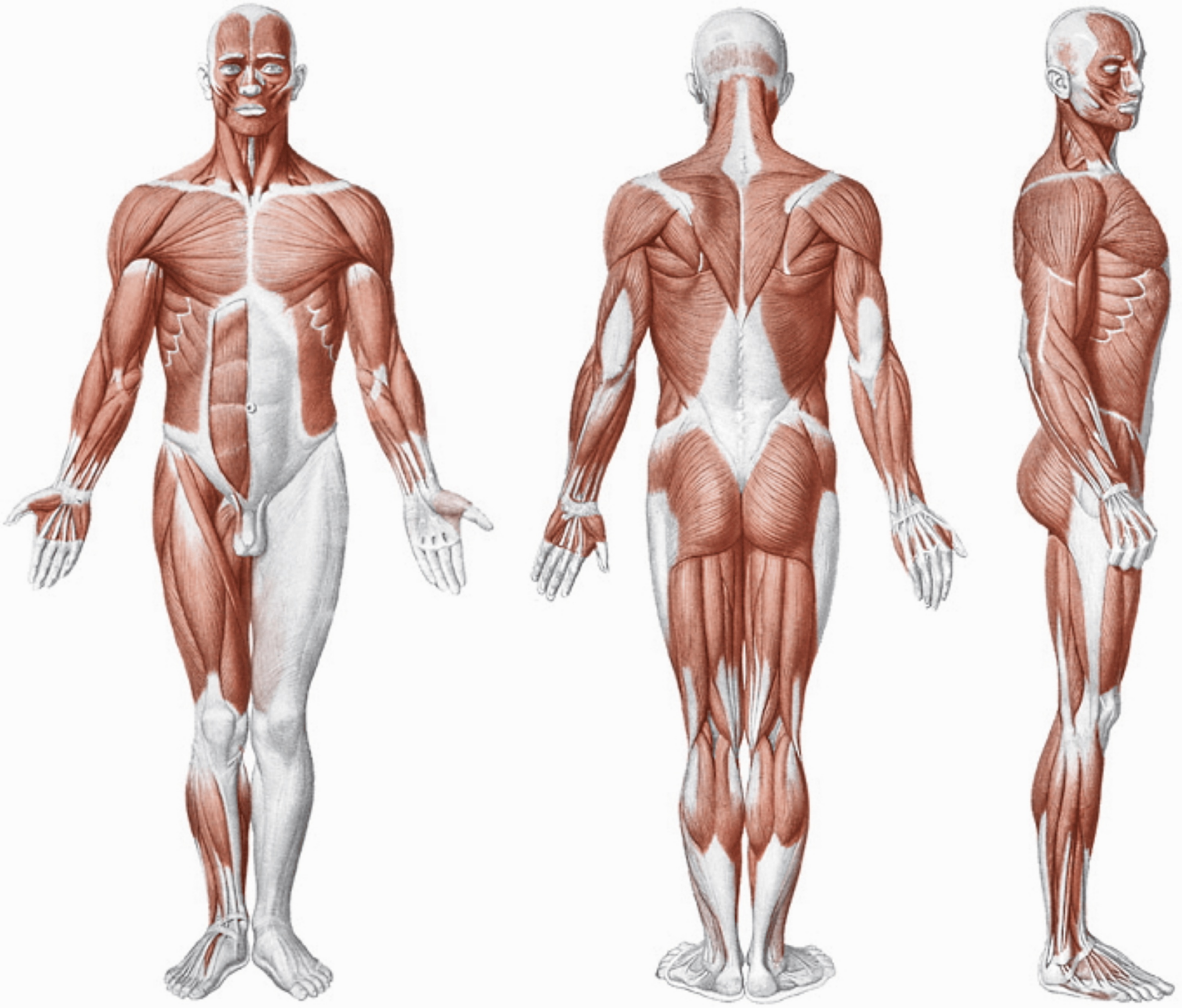
Shape and arrangement of the muscles on the ventral surface (a), dorsal (b) and lateral (c) of the human body. The image resumes the concept of fascial continuity seen only as connective tissue that covers and interpenetrates the muscles. (Reproduced with permission Anastasi et al. AA VV, Anatomia dell'uomo, fourth edition, 2010, pp76. Editor: Edra Edi-Ermes, Milano).

We should leave in the past the mechanistic and reductionist conception that the body is a set of layers. As mentioned in the introductory section, the human body is an absolute continuity, where each tissue is interpenetrated with other tissues and a physiological and non-physiological variable (mechanical, metabolic, chemical, and other) can influence the whole body and not just one segment. An extremely simple example to understand the concept is inflammation. When reading blood values to identify alterations, these alterations reflect the systemic behavior of the body and not only the point where the needle penetrates; the whole body in the presence of a localized trauma, involves the activation of all systems (Figure 1) [38-41].

Defining a tissue

How do you define a tissue? There is a dogma in the literature, that is, to divide tissues into four large categories: epithelial; muscular; nervous; and connective [42]. Despite this categorization, not all the literature is uniform in supporting this vision [42-44].

A tissue (from a histological point of view) is generally identified as a structure of similar cells with a unified function [26]. Some authors highlight that some tissues, such as lymphoid and hematopoietic tissues, dental tissue, and sensory organs do not fall within the previously mentioned classification typologies [42,44]. Furthermore, the same classification does not consider the fact that the tissue functions thanks to other structures that do not originate from the same tissue (vessels, nerves), but in the absence of the latter the function would not exist nor the tissue that they feed [42,44]. Furthermore, we do not fully know every type of cell that constitutes the tissues, and it is not possible to have an adequate understanding of the definition of tissue/cell [43].

We can affirm that not only is there no univocal definition of what is defined as tissue and what is not, but, currently, no one can claim the authority to define what is and what is not connective tissue.

Defining a system

How do you define a system, such as the connective tissue system? In scientific terminology, the term “system” first appears in the sixteenth century [45]. In the second edition of Terminologia Anatomica (2019), the large structures of the human body are separated into systems (muscular system, etc.), but without details on the separation or inclusion of individual structures; for example, the skeletal system includes joints, which are not bones [45]. When classifying a system, several anatomical structures are often included, which from a functional point of view cannot be separated (sympathetic and parasympathetic pathways, which are in both the central and peripheral nervous system) [45].

The set of cells can be considered as a system, and not a static set, but a dynamic system [43]. Therefore, a system can be understood as function and behavior.

In manual fascial medicine, there are still no names and nomenclatures that are specific to manual medicine itself, and that are shared by all operators. Much terminology is taken from anatomical nomenclature, which transition is not always appropriate to the profession performed. Neumann himself writes: “…there are good reasons to hesitate before adopting terms from another field. Not all biomedical disciplines are fortunate enough to have international standard terminologies, such as exist for species, enzymes, genes, drugs and body parts” [45].

We could say that the connective continuum is a system of different tissues, dynamic (it adapts), and static (it maintains its own function and protects that of the tissues it penetrates and envelops).

Defining an organ

How is an organ defined? An organ is a portion of the body made up of multiple tissues, independent or dependent on the rest of the body, which has a vital or special function for maintaining health [46]. In anatomical nomenclature, the term organ can be not only a noun, but also an adjective (“like an organ”); furthermore, an organ can refer to a complex and well-localized structure (liver), or to a larger structure that is found in multiple body areas (skin) [46]. In biology, there is still no agreement on defining what an organ really is; it can be classified based on morphology, function, and embryological derivation [47].

Furthermore, an organ does not have a single function. For example, the heart is known for its circulatory function, but it can secrete hormones (natriuretic peptides) and growth factors (fibroblast growth factors 21, epidermal growth factor, and others), which influence local and systemic metabolism [48-50]. It is the cells (both myocytes and fibroblasts) that constitute the cardiac tissue and that together form it, that influence its functions. The organ itself is made up of cells; it is these that determine the functions of the organ. Another example is skeletal muscle. The latter is recognized as an endocrine organ, thanks to its cellular capacity (striated and connective contractile fibers) to produce in autocrine and paracrine mode, multiple substances (myokines, insulin-like growth factor 1, and others), which influence systemic metabolism [51-53].

Blood is considered an organ, both as a whole and at the cellular level (erythrocytes) [54-56]. Bone tissue is considered an endocrine organ [57,58]. The brain is considered an organ, as are peripheral nerves and ganglia, the spinal cord [59,60]. All tissues can secrete biochemical substances to maintain their own homeostasis and to influence the homeostasis of other tissues (just think of stem cells or different families of cytokines or growth factors). The same principles of reference in biology to define an organ (function, morphology, position, embryology), are used to classify the different types of cells [61]. Each cell communicates with all cells through electromagnetic information, microRNA, influencing each other [1-4]. Probably, it would not be wrong to consider each cell as the mirror of the tissue (shape, function, position, derivation) in fractal mode, and think of the single cell as a small organ. Also, the characteristics of the cell, multiplied from the microscopic to the macroscopic, determine how the tissue behaves and not vice versa.

The fascial continuum, that is, all the tissues that compose the fascia can be considered a set of multifunctional organs. The intellectual difficulty in considering every single part of the body as an interdependent continuum probably lies in the approach deriving from studying the body as a system and not as an organism, observing the body as a district and not as an absolute continuity, or medical and professional hyper-specialization. Each vital unit, tissue, organ, and system is not necessarily a hierarchical organization, but a functional one [47].

A leap into the future

A tissue can be conceived from microscopic boundaries visible only thanks to computer magnification, distinguishing the various layers and tissue levels [26]. This latter is a vision that has persisted since the birth of the microscope.

In reality, the function of living organisms is more complex and cannot necessarily be conceived from a small tissue on a microscope slide. The histological preparation under the microscope is two-dimensional, while the tissue of living organisms is three-dimensional. By observing a cell/tissue with more advanced instrumental techniques, such as holographic flow cyto-tomography, or diffusion tensor magnetic resonance imaging, we can observe functional three-dimensionality [62-64]. Functional three-dimensionality eliminates visible boundaries in a two-dimensional image [65].

To give an example of function, independent of two-dimensional boundaries, is the keratinocyte (epidermis)-fibroblast (dermis) crosstalk. This biochemical relationship allows fibroblasts (thanks to contraction) to activate a mechanotransduction metabolic pathway (Ras: small GTP-binding protein; Raf: serine/threonine-specific protein kinase; ERK: extracellular signal-regulated protein kinase; MEK: specific protein kinase pathways), which stimulates the activation of transcription factors, influencing the gene expression of epidermal maturation; fibroblasts maintain the homeostasis of the epidermis and allow its regeneration [66,67].

In the presence of aberrant scarring, keratinocytes can stimulate the transformation of fibroblasts in the dermis into myofibroblasts, increasing their percentage of detection and aggressiveness (pro-fibrotic positive loop), creating adhesions and fibrosis [68]. Muscle fibers during exercise produce different types of myokines secreted in the paracrine mode for the body system; one of these targets is the skin. It has been shown that the myokine interleukin-15 (through skeletal muscle AMP-activated protein kinase) protects skin aging, probably by protecting the mitochondrial structure of fibroblasts [69,70].

For manual fascial medicine, the hierarchical concept of superficial layers (deriving from 1700) and deep layers does not coincide with the function and has no correspondence with the systemic response, regardless of the area of treatment of the hands, both on animal models and human models [24,71-74]. Furthermore, the decision not to include the epidermal layer in the “superficial” fascia (but starting from the dermis), derives from 1851, and this vision has persisted for centuries, and independently of current knowledge [24]. The future of manual fascial medicine should not be based on layers, districts, or two-dimensionality, but on the three-dimensional body fascial continuum; not on the nomenclative hierarchy but on the systemic function. The functional classification of tissue should be based on the embryological origin, which concept is present as a principle of anatomical nomenclature, but little applied; furthermore, to understand the human body, information that also comes from quantum biological medicine should be used.

Embryological derivation

To identify a tissue function, it is necessary to know its embryological origin beforehand [75,76]. This knowledge allows us to avoid confusion when trying to classify fascial tissue. Several authors, when discussing fascial layers, do not distinguish between the embryological origin of the cranial area, part of the cervical tract, and the rest of the body [26,77]. The connective tissue that separates the different muscular components as regards the cranial area and part of the cervical tract derives from the ectodermal layer, while the same connective tissue that involves the remaining musculature derives from the mesodermal layer [23,78-81].

The matter becomes more complicated for some muscles, as they have a double phylogeny (ectoderm and mesoderm). For example, the various connective septa that separate the muscular complexity of the tongue derive from the ectoderm; the connective tissue within muscles such as the sternocleidomastoid and trapezius derive from the ectoderm and mesoderm; the connective septa found in the skeletal muscles of the trunk and limbs derive from the mesoderm [79,82].

During morphogenesis, mesodermal cells express greater motility than ectodermal cells [83]. Another difference between a tissue of mesodermal and ectodermal origin is the greater repair capacity of ectodermal tissues. From recent data, stem cells (found throughout the body) originating from the ectodermal layer possess a greater repair and adaptation capacity [84,85]. We could hypothesize that, from the point of view of fascial manual medicine, the fascia deriving from the ectoderm is more malleable and receptive; a hypothesis that remains to be verified.

Quantum biology

Can living beings be observed and understood not only by traditional sciences such as medicine but also by quantum biology (or quantum life science)? Quantum biology can be defined as the study of the application of the laws of quantum physics to those aspects of biology that cannot be exhaustively described by the laws of classical physics. As previously stated, the human being is a coherence of structures and reactions that arise from the nanoscopic or quantum world: “Every chemical process relies on quantum mechanics” [86].

For example, the transport of electrons from one protein to another, distant or nearby, through simple vibration of each cellular structure can occur thanks to the phenomenon known as quantum tunneling, where transport does not require energy, it is an extremely rapid action, modulating the cellular and tissue metabolic environment [86,87]. This last mechanism could be the basis of a biological reaction such as the transmission of odors to the olfactory nerve [86,87]. The simple recognition between molecules, the functional expression of cells in tissues up to the functioning of DNA, derives from quantum reactions: “all this is quantum physics and a natural basis for life and everything we see” [88]. A revolutionary application starting from this quantum property (quantum tunneling) is scanning tunneling microscopes (STMs), which are devices used to obtain very high-resolution images of the surface of materials at the atomic level and to observe cellular behavior. They are the basis of the development of nanotechnology and materials science.

Every change (mechanical, chemical, electrical) in the morphology of the cell and its cytoplasmic components causes vibrations; these will generate local and distant electromagnetic fields (waves). Electromagnetic waves are made up of phonons and photons [2,3,89,90]. Every cell in the human body is aware of what is happening in all cells, thanks to these magnetic waves that pervade the entire body at a speed greater than the speed of neural electrical activity [1,4]. Hydrogen atoms, the glue that holds the two strands of the DNA double helix together, can, under certain conditions, behave like waves and thus can exist in more than one position at a time. This means that these atoms can occasionally find themselves on the wrong side of the DNA strand, thus leading to the appearance of mutations. This concept is also based on quantum superposition, which is a principle that describes the possibility that a quantum system can exist simultaneously in multiple distinct states. In other words, while in classical physics an object is in a well-defined state, in quantum physics an object can be in a superposition of states. Recall that in physics the “state” of a particle consists of all the information that can describe it completely at a given moment. In quantum mechanics, the state of a particle is described by a wave function that contains information on the probabilities of finding the particle in different positions or with different physical properties.

From the biological quantum perspective, there are no layers or distances: “living systems are fundamentally quantum mechanical since the dynamics of their molecular, atomic and sub-atomic chemical machinery is, like everything else, governed by the law of quantum physics” [91].

Breathing itself (a systemic and non-local act) generates electromagnetic waves in the neural tissues directly and indirectly involved by the respiratory rhythm, which waves will involve the whole body, from molecular reactions to macroscopic functions [91-94].

From the concept of quantum biology, new medical technologies (nanomedicine) are being born: “All living systems are made up of molecules, and fundamentally, all molecules are described by quantum mechanics” [95]. To give an example, quantum technology-based hyperpolarized MRI/NMR can define three-dimensional molecular behavior and, in the future, identify the best subjective pharmacological spectrum for a specific pathology [95].

The fascial continuum (made up of molecules) cannot be framed in a two-dimensional and hierarchically stratified context but as a constant sub-atomic interaction (quantum coherence) with respect to the macroscopic tissue function (quantum de-coherence). Quantum de-coherence is the cessation of quantum phenomena when such concepts are brought back to the macroscopic. Furthermore, it is not possible to believe in an action that reverberates only in the manual treatment area; regardless of the anatomical area of treatment of the therapist's hands, the whole body and the fascial continuum will create local and systemic adaptations [96-98].

Components of the fascial continuum

Our research group (Foundation of Osteopathic Research and Clinical Endorsement - FORCE) founded in 2013 has published multiple articles on fascial nomenclature, with the perspective of fascial manual medicine [99]. We consider two major nomenclature subdivisions: solid fascia and fluid fascia. This consideration reflects the embryological (and then anatomical) information that we have on the different tissues. For readers who want further information, we recommend reading the articles published on the subject [23,78-81,100-106].

**Figure 2:**
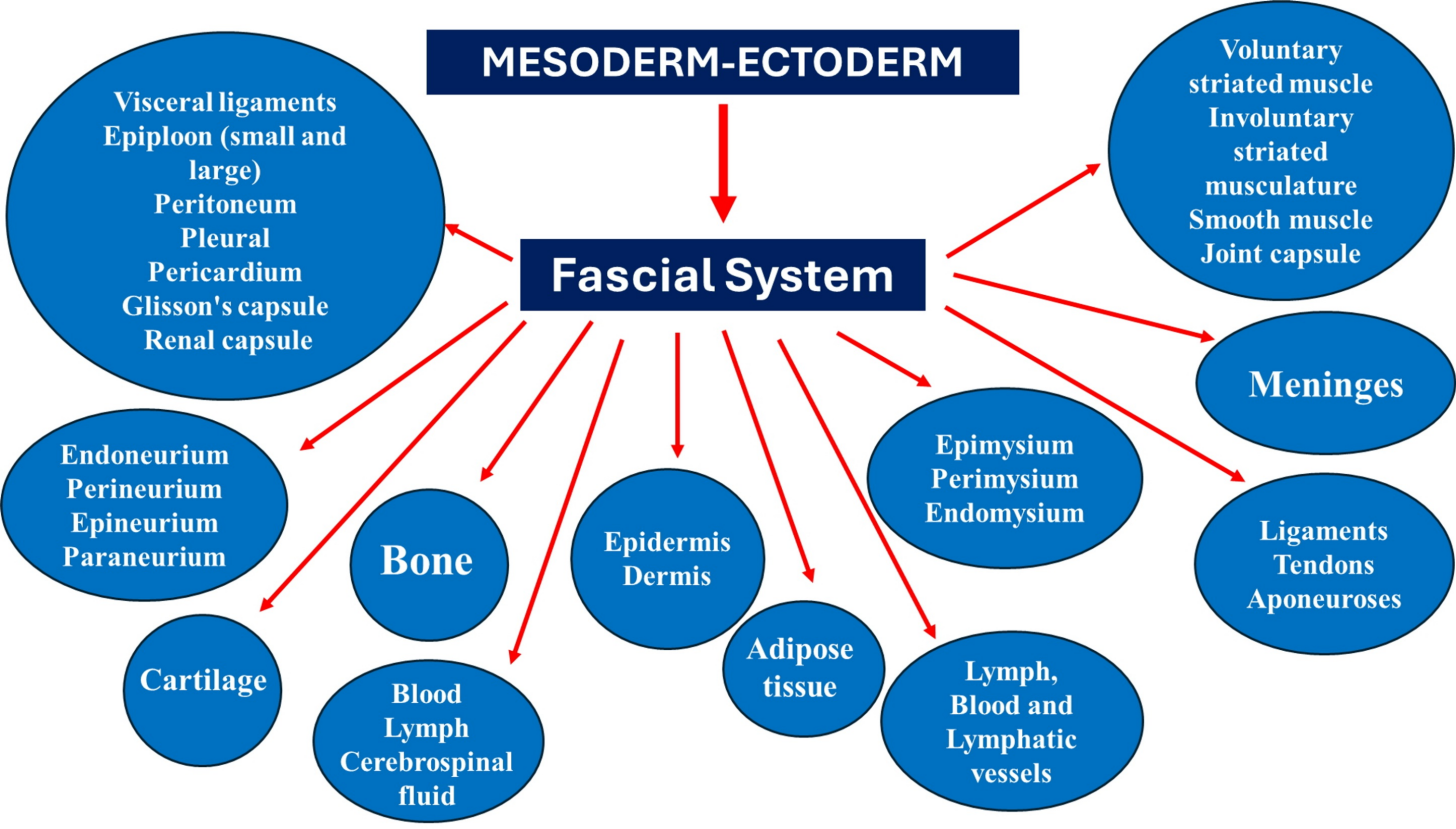
The diagram illustrates what our non-profit organization (FORCE) considers connective tissue. FORCE: Foundation of Osteopathic Research and Clinical Endorsement. The image was created and owned by Bordoni Bruno.

Compared to the nomenclature given by the Fascia Research Congress committee, our denomination of the components that constitute the fascial continuum is:

“The fascial continuum is the result of the evolution of the perfect synergy among different tissues, liquids, and solids, capable of supporting, dividing, penetrating, feeding, and connecting all the districts of the body: epidermis, dermis, fat, blood, lymph, blood, and lymphatic vessels, cerebrospinal fluid, the tissue covering the nervous filaments (endoneurium, perineurium, epineurium, paraneurium), voluntary striated muscle fibers and the tissue covering and permeating it (epimysium, perimysium, endomysium), ligaments, tendons, aponeurosis, cartilage, bones, joint capsule, meninges, involuntary striated musculature and smooth muscle (all viscera derived from the mesoderm), visceral ligaments, epiploon (small and large), peritoneum, pleura, pericardium, Glisson’s capsule, kidney capsule. The continuum constantly transmits and receives mechano-metabolic information that can influence the shape and function of the entire body” (Figure 2) [23].

We could summarize the concepts related to fascial continuity expressed in the article as: the fascial continuum is a system of different non-discontinuous tissues, dynamic and static; all the tissues that make up the fascia can be considered a set of multifunctional organs and can be understood as a sub-atomic coherence that is expressed in the macroscopic function. Most of the self-references cited in the text are articles published in Cureus, which articles represent the vision of our study group (FORCE), and we are in line with the articles of other authors who publish many self-citations on the same topic (but with a different perspective to FORCE).

## Conclusions

The human body acts as a unit, expressing in the macroscopic the functions that derive from the coherence of nanoscopic structures, such as phonons and photons. This narrative review has revised the fascial continuum (made up of molecules), which cannot be framed in a two-dimensional and hierarchically stratified context, but as a constant sub-atomic interaction (quantum coherence) with respect to the macroscopic tissue function (quantum de-coherence). Quantum de-coherence is the cessation of quantum phenomena when such concepts are brought back to the macroscopic. Furthermore, it is not possible to believe in an action that reverberates only in the manual treatment area; regardless of the anatomical area of treatment of the operator’s hands, the whole body and the fascial continuum will create local and systemic adaptations. Further efforts by scientific research are needed to better understand the meaning and functions of fascia, as clinicians who turn to fascia manual medicine must have greater awareness of the local and systemic therapeutic potential.
